# High-latitude ocean habitats are a crucible of fish body shape diversification

**DOI:** 10.1093/evlett/qrae020

**Published:** 2024-05-08

**Authors:** Michael D Burns, Sarah T Friedman, Katherine A Corn, Olivier Larouche, Samantha A Price, Peter C Wainwright, Edward D Burress

**Affiliations:** Department of Evolution & Ecology, University of California, Davis, Davis, CA, United States; Department of Evolution & Ecology, University of California, Davis, Davis, CA, United States; Department of Biological Sciences, Virginia Polytechnic Institute & State University, Blacksburg, VA, United States; Department of Biology and Biochemistry, University of Houston, Houston, TX, United States; Department of Biological Sciences, Clemson University, Clemson, SC, United States; Department of Evolution & Ecology, University of California, Davis, Davis, CA, United States; Department of Biological Sciences, University of Alabama, Tuscaloosa, AL, United States

**Keywords:** latitudinal diversity gradient, ecological opportunity, evolutionary rates, MuSSCRat, disparity, linear morphometrics

## Abstract

A decline in diversity from the equator to the poles is a common feature of Earth’s biodiversity. Here, we examine body shape diversity in marine fishes across latitudes and explore the role of time and evolutionary rate in explaining the diversity gradient. Marine fishes’ occupation of upper latitude environments has increased substantially over the last 80 million years. Fishes in the highest latitudes exhibit twice the rate of body shape evolution and one and a third times the disparity compared to equatorial latitudes. The faster evolution of body shape may be a response to increased ecological opportunity in polar and subpolar oceans due to (1) the evolution of antifreeze proteins allowing certain lineages to invade regions of cold water, (2) environmental disturbances driven by cyclical warming and cooling in high latitudes, and (3) rapid transitions across depth gradients. Our results add to growing evidence that evolutionary rates are often faster at temperate, not tropical, latitudes.

## Introduction

The latitudinal diversity gradient, seen as a strong decline in diversity from the equator to the poles, is one of Earth’s most dominant biodiversity patterns. Although studies on the latitudinal diversity gradient have traditionally focused on patterns of species richness, recent research has also looked at how trait diversity varies with latitude as another dimension of diversity (Drury et al., 2021; Freeman et al., 2022; Wiens, 2023). One hypothesis to explain the greater diversity in the tropics includes greater time for species and morphological diversity to evolve due to a more stable biome (Fischer, 1960; Hawkins et al., 2006; Mittelbach et al., 2007; Wiens & Donoghue, 2004). Other hypotheses predict that faster diversification rates underlie the increase in diversity, either because higher temperatures increase metabolism, which accelerates genetic divergence (Salisbury et al., 2012; Schluter, 2016; Schluter & Pennell, 2017), or because there is greater ecological opportunity, with many niche combinations due to more energy, annual productivity, and biotic interactions (Etienne et al., 2019; Fenton et al., 2023; Pigot et al., 2016; Schluter, 2016). Thus, on the one hand, evolutionary dynamics may be relatively muted, but the stability of tropical regions has allowed lineages time to accumulate diversity, while on the other hand, the tropics are seen as a more ecologically productive and dynamic place that supports higher rates of diversification. Studies on a wide range of taxa have supported both perspectives (Egan et al., 2022; Fischer, 1960; Hawkins et al., 2006; Mittelbach et al., 2007; Schluter, 2016; Schluter & Pennell, 2017).

Although explanations of latitudinal diversity gradients focus on faster evolutionary rates at lower latitudes, others have hypothesized that higher rates should occur in areas with low species diversity (Freeman et al., 2022; Lawson & Weir, 2014; Schluter, 2016; Schluter & Pennell, 2017). Evolution may be the fastest where species diversity is lowest because increased biotic interactions in species-rich regions may limit ecological opportunity and constrain divergence (Freeman et al., 2022; Lawson & Weir, 2014; Schluter, 2016; Schluter & Pennell, 2017). Under this scenario, morphological and speciation rates should be fastest in species-poor temperate zones and slowest in species-rich tropical zones. In support of these predictions, studies across diverse groups have shown that speciation rates and rates of trait diversification are substantially faster in temperate latitudes (Freeman et al., 2022; Lawson & Weir, 2014; Schluter & Pennell, 2017; Weir & Wheatcroft, 2010). While not initially intuitive, this pattern of high speciation and trait evolution rates in relatively depauperate high-latitude areas raises the possibility that these regions, precisely because they have fewer species and biotic interactions, offer conditions that favored rapid adaptive diversification in the recent past (Rabosky et al., 2018; Schluter & Pennell, 2017).

Marine fishes are a particularly interesting group concerning the latitudinal diversity gradient. They strongly exhibit the expected pattern in species richness (Rabosky et al., 2018), with vastly more species in the tropics than at high latitudes. However, speciation rates are significantly higher in high-latitude lineages (Rabosky et al., 2018), suggesting that ecological opportunity might be higher in temperate latitudes. Moreover, a recent study associated high speciation rates in high latitudes with repeated diversification along a depth gradient (Friedman & Muñoz, 2023). Elevated speciation rates in high-latitude marine fishes might also reflect a recent and ongoing poleward expansion of marine diversity (Miller et al., 2018; Rabosky et al., 2018), one which includes some notable adaptive radiations, such as icefishes and rockfishes (Kolora et al., 2021; Near et al., 2012).

In the present study, we explore the nature of marine fish phenotypic diversification across latitudes. Using a phylogenetic approach, we estimate the history of latitudinal occupation by marine teleost fishes and then characterize latitudinal gradients in body shape diversity and the rate of body shape evolution. The expectation and general impression that tropical regions contain greater phenotypic diversity is common in the literature (Rabosky et al., 2018; Ricklefs, 2012) but is inconsistently observed in empirical studies of various groups (Hipsley et al., 2014; Roy & Martien, 2001; Stuart-Smith et al., 2013). These conflicting results indicate that the relationship between species richness and phenotypic diversity is complex, and areas of high species richness are not always more morphologically diverse (Hipsley et al., 2014; Ricklefs, 2012; Roy & Martien, 2001).

Coral reefs and other tropical marine habitats contain many fish species (Rabosky et al., 2018; Stuart-Smith et al., 2013) and high amounts of phenotypic diversity (Price et al., 2019), reflecting adaptations to habitat (Friedman et al., 2020; Price et al., 2013), trophic ecology (Corn et al., 2022; Friedman et al., 2016), and mode of locomotion (Friedman et al., 2021; Larouche et al., 2020). Nevertheless, temperate lineages have also evolved notable morphological variation despite lower species richness. Some temperate lineages exhibit diversification dynamics linked to increased ecological opportunity due to fluctuations in nearshore habitats during oscillations in global temperature (Eastman, 1991; Ingram, 2015; Parker et al., 2022). Furthermore, polar oceans and the temperate North Pacific are home to several radiations that display elevated rates of speciation and morphological evolution (Ingram, 2015; Near et al., 2012; Parker et al., 2022). Thus, both tropical and temperate latitudes may facilitate exceptional radiations of form, but the overall trend in body shape diversity is unclear.

We assess the latitudinal gradient in body shape diversity across 3,183 species of marine fishes. We ask whether differences in time for morphological evolution or rates of evolution provide a better explanation for the observed gradient in body shape diversity. We examine how latitudinal occupation varied through time and whether assemblages reveal a latitudinal diversity gradient. We examine the underlying cause of the latitudinal gradient in body shape diversity in the context of the history of occupation of the latitude quartiles and the rate of body shape evolution in each region. Our macroevolutionary study shows that marine fishes have rapidly expanded their occupation of high-latitude regions in the past 80 million years and experienced a coincident acceleration of body shape evolution. Together with the previously shown elevated speciation rates, our results suggest a strong response to elevated ecological opportunity throughout the Cenozoic.

## Methods

### Data acquisition

Body shape data for marine teleost fishes were taken from a previously published collection of measurements made from museum specimens (Price et al., 2019, 2022). We trimmed this dataset to 3,183 species that matched a tip in a recent time-calibrated phylogenetic hypothesis for ray-finned fishes (Rabosky et al., 2018) retrieved using the fishtree R package (Chang et al., 2019). We analyzed species averages of eight linear measurements that encapsulate the three dimensions of body shape, including standard length, jaw length, mouth width, body width and depth, caudal peduncle width and depth, and head depth. We log-transformed and size-corrected the linear traits by taking the residuals of phylogenetic regressions on body size implemented with the phyl.resid function in the R package phytools (Revell, 2012). We used the geometric mean of the three major axes of body shape (standard length, maximum body width and maximum body depth) as a proxy for body size (Price et al., 2019).

Latitude range centroids were taken from the (Rabosky et al., 2018) study and, following that study, were grouped into discrete latitude bins of the same number of species based on absolute latitude quartiles. This approach allowed us to have equal taxon sampling between the discrete latitude bins. Data available from the Dryad Digital Repository: https://doi.org/10.5061/dryad.fc71cp4. The lower quartile represented species between 0° and 2.51°, the second quartile between 2.52° and 11.50°, the third quartile between 11.51° and 29.72°, and the upper quartile were species above 29.72° latitude (see Supplementary Table S1).

### Reconstructing latitudinal occupation

To reconstruct the history of latitude occupation, we estimated ancestral states with the R package corHMM, using joint inferences of the ancestral states. We fit alternative versions of the Mk model, one model with equal rates (ER) and another with all rates different, and evaluated the presence of one, two, three, or four rate regimes per state across branches (Beaulieu et al., 2013). We used an unrestricted CTMC that permitted transitions between all quartiles to accommodate cladogenic changes in quartile habitation. Transitions between nonadjacent quartiles were permitted in our dataset as taxa were assigned to quartiles based on the centroid of their occupancy range, and thus, the range of a species may extend beyond the quartile to which it is assigned. Those models were compared using sample size corrected Akaike information criteria (AICc) and Akaike weights (AICw). We then used the best-fit model parameters (Supplementary Table S2) to generate a distribution of 1,000 stochastic character maps using makeSimmap (Beaulieu et al., 2013). We then calculated the proportion of branches at million-year intervals in each latitude quartile to determine the proportion of time spent in each state throughout the last 190 million years. This approach does not consider historical biogeographic processes that may have influenced the mid-range latitude occupation of marine fishes, including the centers of origin for extant lineages or how ranges have shifted during continental drift.

### Estimating latitudinal gradients of morphospace occupation, evenness, and disparity

To determine the primary axes of morphological diversity in marine fishes, we performed a principal components analysis (PCA). We calculated body shape disparity for the assemblage of species found in each latitude quartile as body shape variance using the function morphol.disparity in the R package geomorph (Baken et al., 2021). Disparity was calculated across all axes using the overall mean. We computed the multivariate (matrix of all eight morphological traits) morphological variance for each quartile. To visualize how patterns of disparity varied geographically, we combined morphological disparity with species occurrence data collected by Rabosky et al. (2018). The occurrence data is constructed as a global grid with 150 km × 150 km resolution and details the species present in each cell. We determined the species in our dataset present in each cell and evaluated the disparity for every marine cell for 22,736 cells. These results were then plotted as a heatmap across the globe.

We estimated the extent to which fish in each quartile have filled the morphospace by computing the volume of morphospace occupation in each latitude quartile as the multivariate volume of the minimum polygon. We used the first six axes of the PCA, which accounted for 98.2% of the variance. We then calculated the absolute difference in volume between each pair of quartiles. To assess whether the differences in volume between quartiles were statistically significant, we permuted quartile assignments among our species data 1,000 times and compared the volume among the permutated quartiles. We calculated the number of replicates in which the permutated difference in volume was greater than the empirical difference in volume. Finally, we divided this number by the total number of replicates to determine whether the differences in the empirical volume between quartiles were significant.

We estimated the functional evenness to describe the regularity of the distribution of species (and their abundances) in trait space (Villéger et al., 2008) using the fundiversityR package (Grenié & Gruson, 2023). We used the first six axes of the PCA to calculate the overall functional evenness, as well as each principal component individually. We then calculated the difference in functional evenness between each pair of quartiles. To assess whether the differences in functional evenness between quartiles were statistically significant, we permuted quartile assignments among our species data 1,000 times and compared the functional evenness among the permutated quartiles. We then calculated the number of iterations in which the permutated difference in functional evenness was greater than the empirical difference in functional evenness. Finally, we divided this number by the total number of iterations to determine whether the differences in the empirical functional evenness between quartiles were significant.

The dispersion of taxa in a morphospace can be biased by how many taxa are sampled within each clade and which specific taxa are measured from those clades. To determine whether the various species sampled from each clade might be biasing our estimates of morphological variance, volume, and evenness, we performed a bootstrap analysis 1,000 times for the different dispersion metrics. We qualitatively compared the distribution of bootstrapped values to the empirical values for each analysis. Bootstrapping the empirical data allows us to determine whether the different analyses of morphological dispersion are robust to any potential bias in taxon sampling.

### Evolutionary rates

To evaluate rates of body shape evolution across the latitudinal gradient, we employed a modified rate-by-state test (Reynolds et al., 2016). First, we estimated tip rates (i.e., species-specific rates) using a relaxed-clock multivariate model of evolution following (Burress et al., 2020), implemented in RevBayes (Höhna et al., 2016). We used a random local clock model to estimate the rates. The Markov chain Monte Carlo (MCMC) was run for 300,000 iterations with 10% burnin. We then regressed the tip rates against latitude and absolute latitude midpoints. We assessed the correlation between tip rates and mid-range latitude using a phylogenetic regression with robust M-estimation (Revell, 2010). We used a robust M-estimation because it is less sensitive to outliers, and our tip rates contain outlier species. The robust phylogenetic regression was done using the R package ROBRT (Adams et al., 2023). Furthermore, we plotted the geographic distribution of tip rates using the occurrence data collected by Rabosky et al. (2018) following the methodology outlined above. Tip rates for each geographic region were calculated as the average tip rate of the species inhabiting each 150 km × 150 km grid.

To further test the relationship between rates of body shape evolution and latitude, we employed a state-dependent, uncorrelated log-normal (UCLN) model of evolution (Drummond et al., 2006; May & Moore, 2020), implemented in RevBayes (Höhna et al., 2016). The MuSSCRat model jointly estimates the evolutionary history of the discrete and continuous characters and accommodates background rate variation (i.e., rate heterogeneity not attributable to the focal discrete character), thereby reducing biases in rate estimates (Burress & Muñoz, 2022; May & Moore, 2020; Revell, 2013). As a discrete character, we used latitudinal quartiles as described above, and as continuous characters, we used the eight size-corrected body shape traits. The MCMC was run for 300,000 iterations with 10% burnin. To evaluate the sensitivity of posterior parameter estimates, we repeated the MCMC across different priors on the number of rate shifts (400, 500, and 600 shifts).

## Results

### Evolutionary history of latitudinal occupation

**Figure 1:**
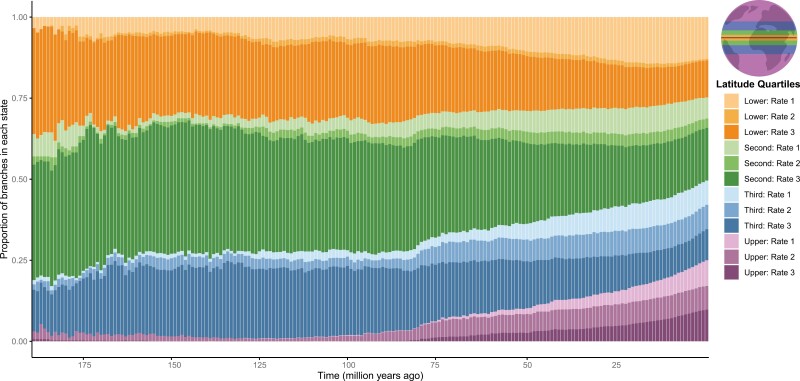
A bar plot showing the proportion of branches at million-year intervals in each absolute latitude quartile generated from the ancestral state reconstruction using stochastic character mapping. The three rate categories in each quartile correspond to hidden rate categories, ranging from the lowest transition rate (Rate 1) to the highest (Rate 3). The lower quartile represented species between 0° and 2.51°, the second quartile between 2.52° and 11.50°, the third quartile between 11.51° and 29.72°, and the upper quartile were species above 29.72° (see inset map for quartile locations).

The stochastic character maps indicate a relatively stable occupation of all latitude quartiles from the base of the phylogeny, about 190 Mya until about 80 Mya, when the upper quartile (including polar, subpolar, and temperate fishes) started to increase in proportion (Figure 1). Prior to about 80 Mya, the lower, second, and third quartiles were occupied by about 90% of extant marine teleost lineages, with only about 10% of lineages occurring within the upper quartile. Beginning shortly before the end-Cretaceous, the proportion of lineages occupying upper quartile latitudes steadily increased until reaching its peak today at about 25% of branch length in the past million years.

### Body shape evolution and disparity across latitude

**Figure 2:**
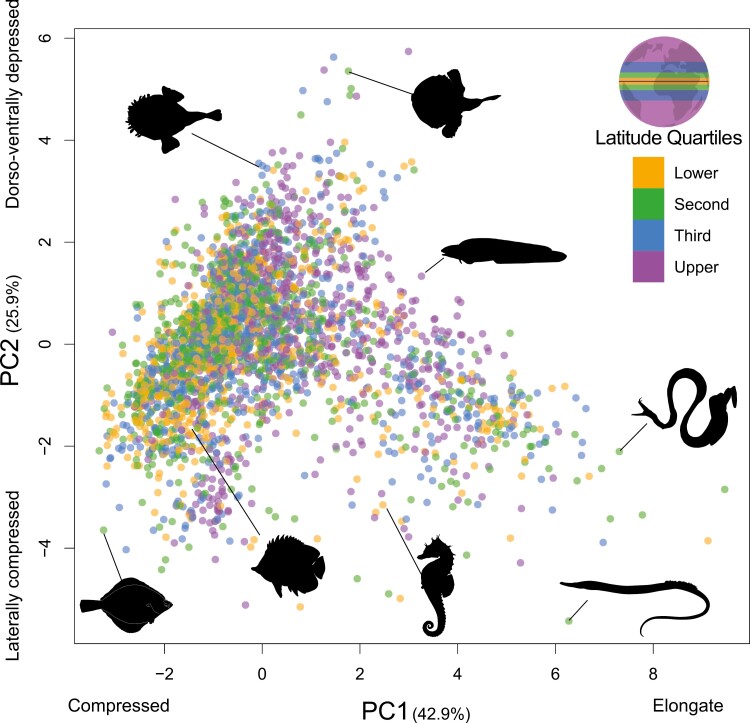
Major axes of body shape variation in 3,183 species of marine teleost fishes, represented by the first two principal component axes (PCs) from a principal components analysis on eight linear traits. Each point is the average shape of a species, colored by the absolute latitude quartile. Drawings show representative species illustrating morphological variation across the plot. The lower quartile represented species between 0° and 2.51°, the second quartile between 2.52° and 11.50°, the third quartile between 11.51° and 29.72°, and the upper quartile were species above 29.72° (see inset map for quartile locations).

Body shape varies substantially in marine fishes (Figure 2). Species in each latitude quartile are found in all regions of the morphospace, with marine fishes evolving shapes that range from highly elongate bodies, those that are dorsoventrally compressed, and others that are laterally compressed, regardless of latitude. The volume of morphospace occupied differed slightly between the quartiles, but only the upper and second quartiles exhibited significant differences (Supplementary Figure S1 and Supplementary Table S3). The volume of morphospace occupied by each quartile was similar when the data were bootstrapped (Supplementary Figure S2). The distribution of species occupying each quartile showed qualitative variation. Species in the lower, second, and third latitude quartiles are concentrated in an oval-shaped region of morphospace distributed in the lower half of PC1 and PC2, represented by mostly laterally compressed, deep body shapes. While species in these latitudes evolved into all other regions of morphospace, these body shapes are represented by fewer species. In contrast to the other latitude quartiles, body shapes of upper quartile species are significantly more evenly distributed across Principal Component 1 (Supplementary Table S4), with similar results when the data was bootstrapped (Supplementary Figure S3). Most notable is the concentration of species with elongated, somewhat wide body shapes in the upper quartile, a morphotype that is more poorly represented in the other quartiles (Figure 2).

**Figure 3:**
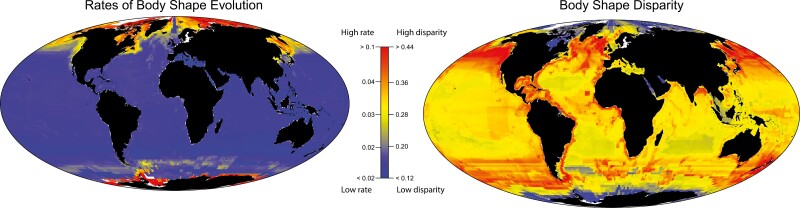
Mean tip rate and disparity of body shape for marine fish assemblages at the global scale. Cell colors correspond to the scale bar, with warmer colors representing higher rates of morphological evolution and morphological disparity. Grid cell size is 150 km × 150 km for both images.

These impressions of distributions within morphospace were confirmed by the disparity of multivariate body shape, which was highest in the upper quartile compared to the other latitude quartiles (Table 1), though significant differences only occurred between the upper and lower quartiles. No other latitude quartile showed significant differences in body shape disparity, although the trend is for increasing disparity as one moves away from the equator (Table 1; Figure 3). However, when disparity is viewed on a finer geographic scale, we see variation in patterns of disparity within each latitudinal quartile (Figure 3). Some regions close to the equator had high levels of disparity, including the west coast of Africa and the eastern portion of the Neotropics (Figure 3), while some regions near the poles had low disparity. When bootstrapping the data, we see that body shape disparity was consistently higher in the upper quartile compared to the other latitude quartiles (Supplementary Figure S4). These results suggest that the effect of latitude on body shape evolution is particularly acute in the upper quartile.

**Table 1:** Morphological variance and pairwise differences between variances for a multivariate matrix of all eight traits for each latitude quartile.

	**Lower**	**Second**	**Third**	**Upper**
**Procrustes variance for each group**				
Procrustes variance	0.276	0.312	0.322	0.349
**Pairwise absolute difference between variances**				
Lower	–	0.036	0.046	0.073*
Second		–	0.009	0.037
Third			–	0.027
Upper				–

*Note*. Asterisks represent significant differences at *p* < 0.05. The lower quartile represented species between 0° and 2.51°, the second quartile between 2.52° and 11.50°, the third quartile between 11.51° and 29.72°, and the upper quartile were species above 29.72°.

### Rates of body shape evolution across latitude

**Figure 4:**
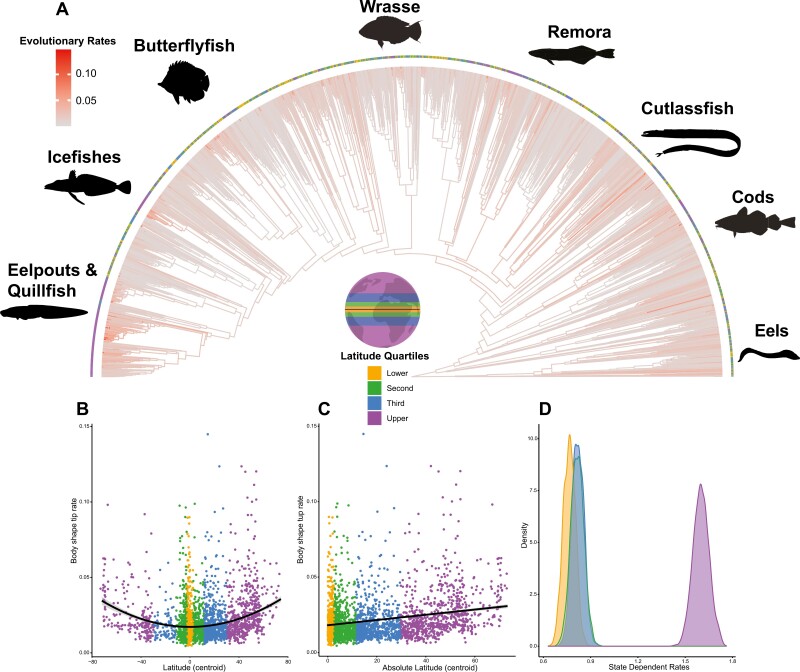
(A) Branch-specific rates of morphological evolution across a phylogenetic tree of marine teleost fishes. Warmer colors represent faster rates of evolution. Colored circles at the tip represent latitude quartiles. There is a significant relationship between morphological evolutionary rate, (B) latitude, and (C) absolute latitude. (D) Density plot of state-dependent rates of body shape evolution estimated with a Bayesian, state-dependent, relaxed-clock model of multivariate Brownian motion. The lower quartile represented species between 0° and 2.51°, the second quartile between 2.52° and 11.50°, the third quartile between 11.51° and 29.72°, and the upper quartile were species above 29.72° (see inset map for quartile locations).

Rates of body shape evolution varied among marine fishes (~31-fold), including elevated rates in eelpouts (Zoarcidae), icefishes and their allies (Notothenioids), and cods (Gadidae) (Figure 4A). A phylogenetic regression shows that the rates were significantly but weakly correlated with latitude (Figure 4B; *R*^2^ = 0.13; *p* < 0.05) and absolute latitude (Figure 4C; *R*^2^ = 0.12; *p* < 0.05). The rate of body shape evolution was state-dependent (Posterior Probability > 0.95), with the fastest rates occurring in the highest latitudes (Figures 3 and 4D). This result was consistent across replicates with different priors (all P *p* > 0.95). The upper latitude quartile had faster evolutionary rates than the lower (2.1-fold), second (1.9-fold), and third (1.9-fold) quartiles (Figure 3D).

## Discussion

Body shape varies spectacularly in marine fishes, but the diversity of body shapes is not spread evenly between the equator and the poles. Although the tropical lower latitudes house vastly more species, we find that the disparity of body shape is greatest and is evolving fastest at mid to high latitudes. We also show that the occupation of upper-latitude oceans has increased substantially in the past 80 million years. Together with the dramatic environmental changes accompanying the cyclical warming and cooling that polar oceans undergo, these results suggest that the evolution of fishes in these regions has fluctuated throughout the Cenozoic. Our results add to the growing body of evidence that the poleward expansion of organisms during Earth’s most recent deglaciation has often led to more rapid evolution in the temperate zone than in the tropics.

Occupation of upper latitudes by extant marine lineages was relatively rare until an ongoing invasion that began in the late Cretaceous. Upper latitude occupation began increasing around 80 Mya, a period when many major lineages of marine teleosts originated (Ghezelayagh et al., 2022). While our ancestral state reconstructions do not account for historical biogeographic processes such as changes in the center of origins or continental drift, our finding of a recent increase in upper latitude occupation is supported by a previous biogeographical analysis which found that the colonization of high latitude marine habitats increased over the Cenozoic (Miller et al., 2018). This relatively recent history of upper latitude fishes indicates that increased time for morphological evolution is not the driving factor behind their high disparity, and low rates of evolution are not the primary factor behind low disparity among fishes at the poles. Instead, we found that the rate of body shape evolution is substantially faster in latitudes closer to the poles. Rates decrease substantially as species move closer to the Earth’s equator (Figure 3). Increased upper latitude occupation and elevated rates of body shape evolution occur in several major North Pacific and Polar Ocean clades (Figure 1), including the eelpouts and icefishes (Figure 3). Together with previous work (Miller et al., 2018; Rabosky et al., 2018), these results demonstrate that while tropical regions house the greatest marine teleost species diversity, lineages in upper temperate regions are currently experiencing more rapid body shape evolution and speciation.

One classic hypothesis for the latitudinal diversity gradient is that greater ecological opportunity in the tropics, caused by increased productivity and biotic interactions, drives higher rates of species and morphological diversification through character displacement and niche partitioning (Etienne et al., 2019; Fenton et al., 2023; Pigot et al., 2016; Schluter, 2016). In contrast to this viewpoint, others have predicted that ecological opportunity is highest in areas with low species diversity because the decreased biotic interactions result in more open niches (Cooper & Purvis, 2010; Freeman et al., 2022; Weir & Wheatcroft, 2010). Ecological opportunity can increase through the availability of new adaptive zones (Simpson, 1944) following key innovations or entry into new geographic regions with unfilled niches (Givnish, 2015; Schluter, 2000; Simpson, 1953), or a combination of the two (Near et al., 2012; Schluter, 2000). Both mechanisms potentially operated in upper latitude marine fishes. The two groups with the highest rates of phenotypic evolution, the eelpouts and icefishes (Figure 3), contain lineages that independently evolved antifreeze proteins (Deng et al., 2010; Hew et al., 1988; Near et al., 2012). This novelty allowed these groups to exist in seawater that can be a full degree lower than the freezing point of their body fluids, as found in polar and subpolar oceans (Bar Dolev et al., 2016; Fletcher et al., 1986; Raymond & DeVries, 1977) that otherwise contain relatively few other lineages (Eastman, 1991; Hobbs et al., 2020). The evolution of antifreeze proteins allowed these lineages to invade a biome rich in open niches independently and has increased the rates of morphological evolution and speciation in notothenioid icefishes (Dornburg et al., 2017; Near et al., 2012). Furthermore, the extinction of other competitors enabled these ancestrally benthic fishes to invade newly available pelagic foraging niches, resulting in associated morphological diversification (Eastman, 2005; Eastman & DeVries, 1981; Matschiner et al., 2011). However, other upper latitude clades have evolved antifreeze proteins and do not exhibit elevated body shape evolution rates, including snailfishes and sculpins (Fletcher et al., 2001; Gong et al., 1995; Wainwright & Longo, 2017). Thus, the evolution of antifreeze proteins that facilitated access to polar and subpolar oceans in upper latitudes has likely played a role in the high rates of body shape evolution seen in some of the upper latitude lineages, though it is not the only factor influencing the pattern.

Ecological opportunity in polar and subpolar oceans in upper latitudes also may have been impacted by cyclical warming and cooling, resulting in environmental disturbances and reshaping the physical environment across different geologic periods (Clarke & Crame, 2010; Dornburg et al., 2017; Near et al., 2012; O’Loughlin et al., 2011). The creation of novel niches due to environmental disturbance could underlie elevated rates of body shape evolution (Corn et al., 2022; Dornburg et al., 2017; Folk et al., 2019; Lucek et al., 2018; Near et al., 2012; Parker et al., 2022; Rutschmann et al., 2011). For instance, the periodic disturbance of the environment around Antarctica, in which ice scours eliminated habitat during cool periods and habitats reopened during warm periods, has been identified as a key factor behind the repeated ecomorphological diversification in cryonotothenioid icefishes (Parker et al., 2022) and eelpouts (Hotaling et al., 2021). The changing environment in upper latitude biomes may have facilitated the repeated generation of ecological opportunity and promoted the rapid evolution of body shape (Dornburg et al., 2017; Near et al., 2012; Parker et al., 2022; Rutschmann et al., 2011).

Many upper latitude lineages transition across depth gradients more rapidly than lower latitudes (Friedman & Muñoz, 2023), which may increase ecological opportunity and subsequent morphological diversification. For example, eelpouts (Zoarcidae), rockﬁshes (Sebastidae), ﬂatﬁshes (Pleuronectidae), and iceﬁshes and their allies (Notothenioids) transition across depth zones more readily than expected based on clade age and species richness (Friedman & Muñoz, 2023). In our dataset, these same clades exhibit some of the highest rates of body shape evolution. Different depths in marine environments provide multiple axes along which fishes can diversify, including diet, microhabitat, and life history strategies (Dornburg et al., 2017; Gerringer et al., 2021; Kolora et al., 2021). These different axes likely increase the potential for morphological evolution in these clades. For instance, Antarctic ice fishes diversify along the benthic-pelagic axis, with repeated ecomorphological diversification during invasions of pelagic, benthic, semipelagic, and epibenthic niches and exhibit subsequent morphological adaptation to dietary niches in those habitats (Parker et al., 2022; Rutschmann et al., 2011). Furthermore, temperate North Pacific Rockfish exhibit significant morphological diversification related to transitions across depth gradients and diet niches (Ingram, 2015; Ingram & Kai, 2014; Ingram & Shurin, 2009). Repeated diversification across depth gradients in upper latitude biomes may have contributed to the high disparity and rapid evolution of body shape.

Although we might predict that body shape diversity in marine fishes would be higher in tropical lower latitudes following the patterns of species richness instead, we find that the range of body shapes is relatively constant across latitudes while disparity increases in high temperate areas (Figure 2; Supplementary Figure S1). Despite some regions of high disparity in the tropics, often associated with continental edges such as the west coast of Africa and the eastern portion of the Neotropics (Figure 3), the overall pattern of low tropical body shape disparity and high species diversity exists because lower latitude species are more densely packed in morphospace. Species from tropical lower latitude habitats are particularly densely packed in a region of morphospace corresponding to laterally compressed, short, deep-bodied phenotypes (Corn et al., 2022). Coral reefs also house many fusiform or highly elongated species and a few that are very wide-bodied. These forms provide a variety of adaptations to life within highly structured environments (Domenici et al., 2008; Larouche et al., 2020; Webb, 1983) that facilitate precise maneuverability, moving within the sand or inside reef structures, and sustained swimming (Webb, 1982, 1984, 1988). In contrast, upper-latitude fishes often occur in the lower right region of the body shape morphospace (Figure 2). This includes several lineages that have long slender bodies with tapered tails, such as eelpouts (Zoarcidae), snailfishes (Liparidae), and grenadiers (Macrouridae), a body shape shown to perform efficient, slow-speed undulatory swimming (Tytell & Lauder, 2004; van Ginneken et al., 2005) and is also well-represented in the cold waters of the deep ocean (Martinez et al., 2021; Neat & Campbell, 2013). The laterally compressed, deep-bodied shape of actively foraging butterflyfishes, surgeonfishes, and other coral reef groups is much less common at upper latitudes. Instead, there is a greater occurrence of wide-bodied species, indicative of a benthic lifestyle, and the relatively wide, elongated forms that characterize efficient, slow swimming (Figure 2). Thus, water temperature may have pervasive effects on the activity of marine fishes, which at upper latitudes may manifest in more frequent use of body shapes that are adaptive for less active lifestyles due to decreasing metabolic rates and locomotor capacity in colder waters (Castro-Santos et al., 2022).

Some lineages of marine fishes exhibit antitropical distributions where they inhabit temperate or polar habitat patches on either side of the tropics (Briggs, 1987; Hubbs, 1952; Ludt, 2021; Ludt & Myers, 2021). These antitropical distributions have likely influenced patterns of diversification across a latitudinal diversity gradient within a few clades of ray-finned fishes. However, only 35 families of marine ray-finned fishes contain at least one antitropical species (see Hubbs (1952) and Randall (1981) for a review of families that exhibit an antitropical distribution), and the low number of species showing this pattern means antitropical distributions are unlikely to influence the broad patterns observed in our study. However, future studies should explicitly test whether antitropical distributions affect patterns of diversification in marine fishes.

Our study adds to evidence that recent evolutionary rates are often more rapid at upper latitudes than in lower latitudes (Freeman et al., 2022; Lawson & Weir, 2014; Miller et al., 2018; Rabosky et al., 2018; Schluter & Pennell, 2017; Weir & Wheatcroft, 2010). We show that extant marine fishes exhibit faster body shape evolution in upper versus lower latitudes, possibly because of increased ecological opportunity due to open niches, changing environments, and increased transitions across depth gradients. The faster evolution seen in upper latitudes across multiple taxonomic groups might be a shared evolutionary response to ecological opportunities created by the poleward expansion of organisms during Earth’s cyclical warming (Miller et al., 2018; Schluter & Pennell, 2017). The recent rapid evolution at upper latitudes could continue as more lineages expand into warming temperate zones during accelerated climate change. However, the generality of the morphological pattern remains unknown as most studies have focused exclusively on speciation rates or looked at morphological evolution across only a few radiations. Until more studies examine how morphological diversification dynamics vary across latitudes, it will remain unclear whether the patterns uncovered here are general for most of life on Earth or unique to a few exceptional radiations.

## Supplementary material

Supplementary material is available online at *Evolution Letters*.

qrae020_suppl_Supplementary_Tables_S1

qrae020_suppl_Supplementary_Tables_S2-S4_Figures_S1-S4

## Data Availability

Morphological data have been deposited in Dryad (https://doi.org/10.5061/dryad.34tmpg4qf) and scripts are available on Zenodo (https://doi.org/10.5281/zenodo.7803539).
