# Supplementary material for: High-latitude ocean habitats are a crucible of fish body shape diversification
Source: Evol Lett. 2024 May 8;8(5):669–79. doi: 10.1093/evlett/qrae020 (PMC11424081; doi:10.1093/evlett/qrae020)
Supplement: qrae020_suppl_Supplementary_Tables_S2-S4_Figures_S1-S4 [file qrae020_suppl_supplementary_tables_s2-s4_figures_s1-s4.docx]

**
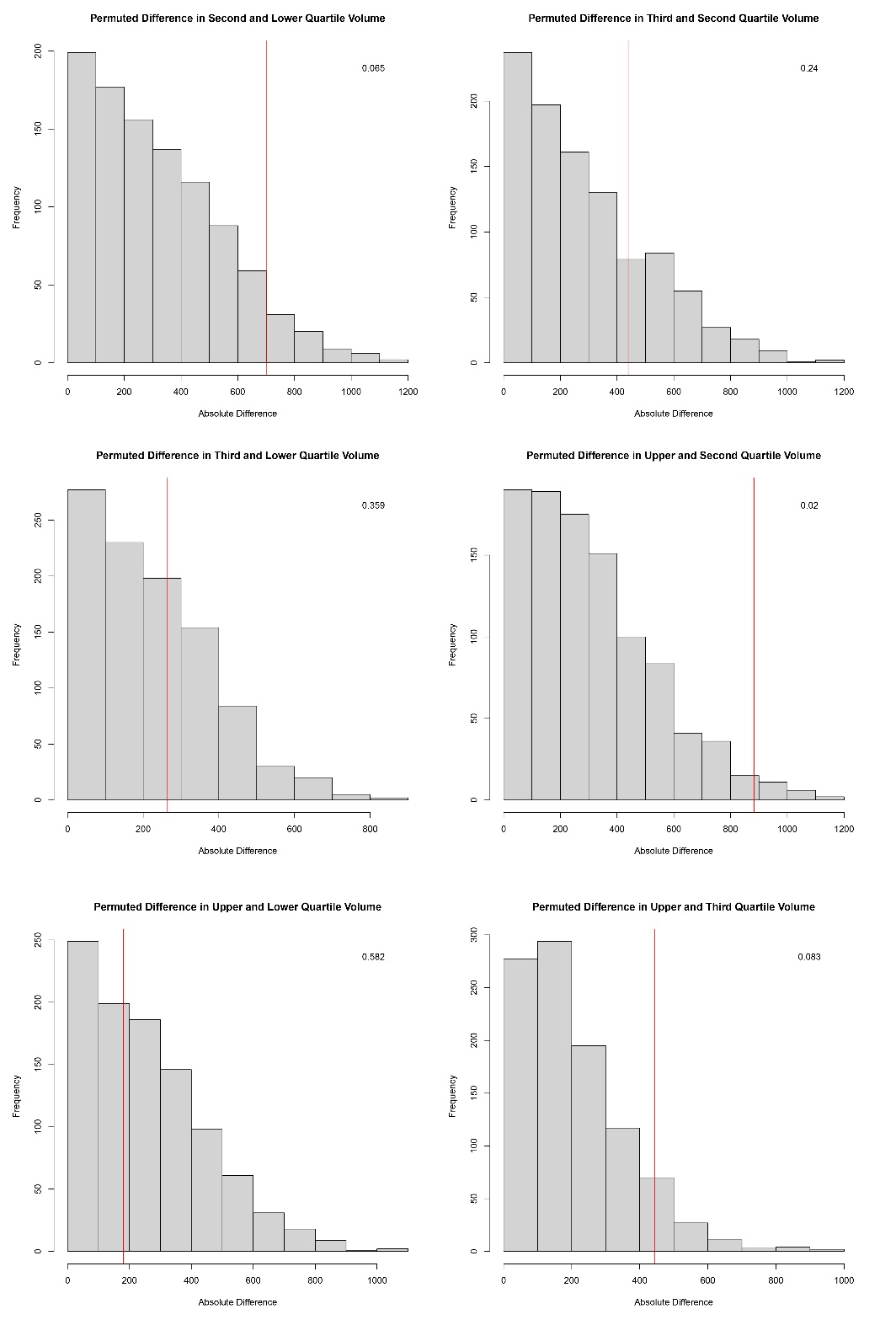
**

**Supplementary Figure S1.** Histograms comparing the absolute difference in volume between each latitude quartile's first six principal components (red line) to the distribution of 1000 absolute difference in volume between permutations of each quartile.

**
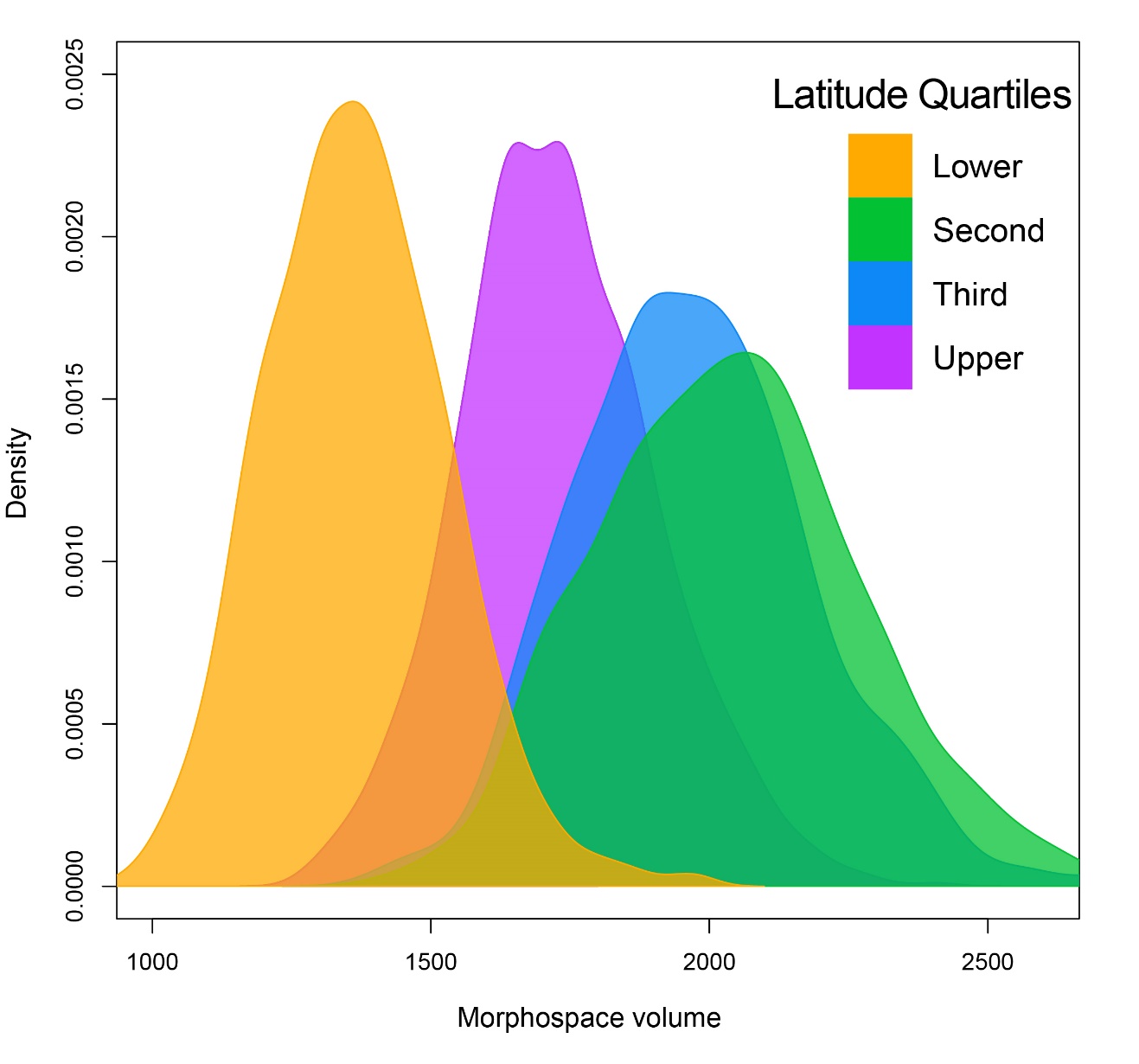
**

**Supplementary Figure S2.** Density plot showing the morphospace volume of each latitude quartile when the data was bootstrapped 1000 times. Lower quartile represented species between 0° and 2.51°, the second quartile between 2.52° and 11.50°, the third quartile between 11.51° and 29.72°, and the upper quartile were species above 29.72°.


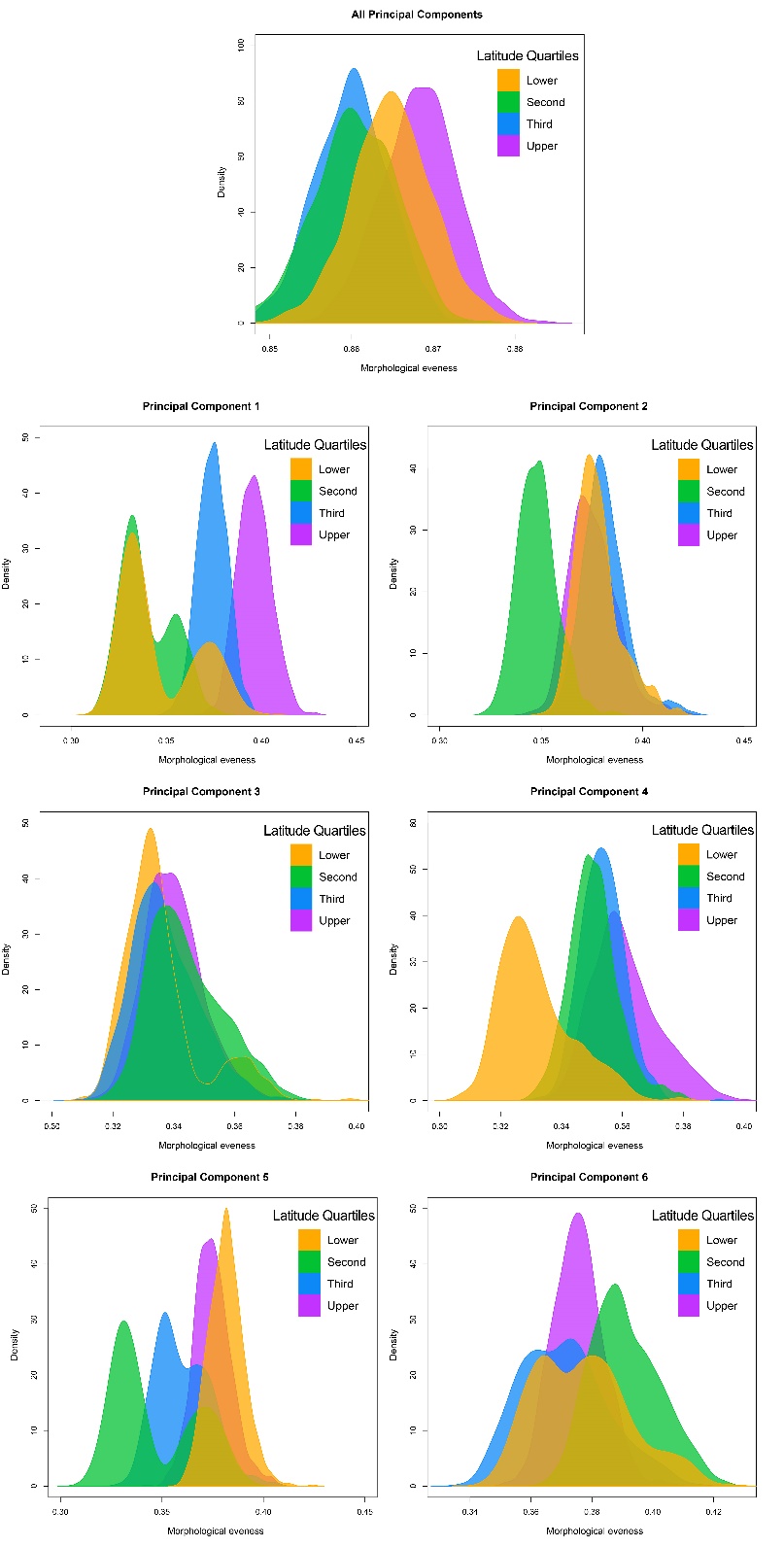


**Supplementary Figure S3.** Density plot showing the morphospace eveness of each latitude quartile when the data was bootstrapped 1000 times. Lower quartile represented species between 0° and 2.51°, the second quartile between 2.52° and 11.50°, the third quartile between 11.51° and 29.72°, and the upper quartile were species above 29.72°.

**
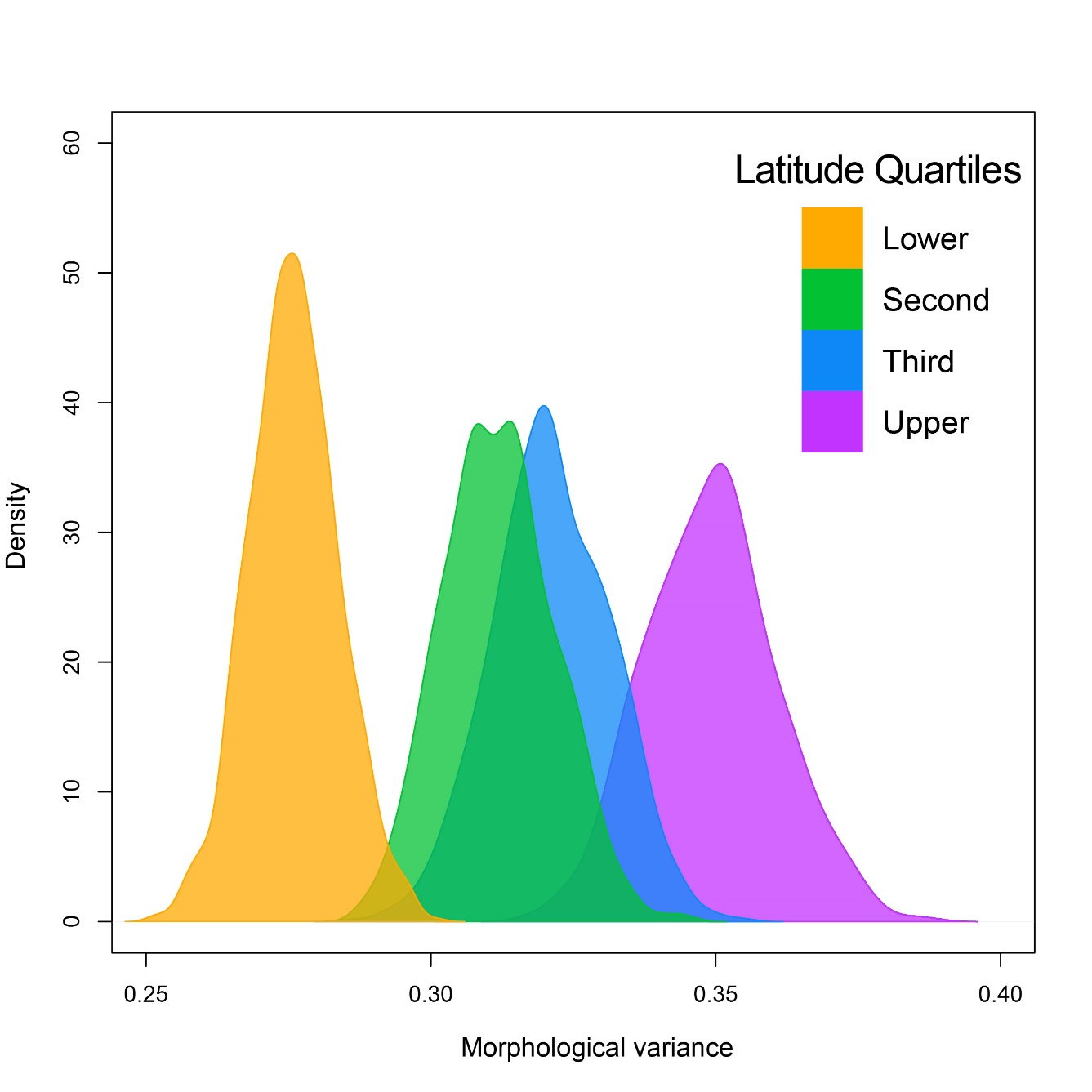
**

**Supplementary Figure S4.** Density plot showing the morphological variance of each latitude quartile when the data was bootstrapped 1000 times. Lower quartile represented species between 0° and 2.51°, the second quartile between 2.52° and 11.50°, the third quartile between 11.51° and 29.72°, and the upper quartile were species above 29.72°.

**Supplementary Table S1.** 3183 species of marine teleosts included in this study, with associated latitude quartile designations.

The table is included as a separate .xlsx file.

**Supplementary Table S2.** Comparison of model fits from corHMM. Parameter estimates from the best-fit model were used for stochastic character mapping.

| Model | AICc | diff | AICw |
| --- | --- | --- | --- |
| ARD: 3 rate categories | 6816 | 0 | 0.92 |
| ARD: 4 rate categories | 6821 | 5 | 0.08 |
| ARD: 2 rate categories | 6900 | 84 |  |
| ARD: 1 rate categories | 7188 | 372 |  |
| ER: 3 rate categories | 7397 | 581 |  |
| ER: 4 rate categories | 7401 | 585 |  |
| ER: 2 rate categories | 7468 | 652 |  |
| ER: 1 rate categories | 7882 | 1066 |  |

**Supplementary Table S3.** Total volume and pairwise absolute differences between volumes for each latitude quartile's first six principal components. Asterisks represent significant differences at P < 0.05.

| **Total Volume** | | | | |
| --- | --- | --- | --- | --- |
|  | Lower | Second | Third | Upper |
| Volume | 1436.35 | 2138.45 | 1698.99 | 1255.67 |
| **Pairwise absolute difference between volumes** | | | | |
|  | Lower | Second | Third | Upper |
| Lower | - | 702.1 | 262.65 | 180.68 |
| Second |  | - | 439.46 | 882.78 |
| Third |  |  | - | 443.33 |
| Upper |  |  |  | - |

**Supplementary Table S4.** Functional evenness for each latitude quartile's first six principal components and each principal component. Asterisks represent a significantly higher functional evenness for the upper latitude quartile versus the other quartiles at P < 0.05.

|  | Lower | Second | Third | Upper |
| --- | --- | --- | --- | --- |
| All | 0.8013* | 0.8033* | 0.8178* | 0.8406 |
| PC1 | 0.4103* | 0.4101* | 0.4781 | 0.5020 |
| PC2 | 0.4619 | 0.4233 | 0.4772 | 0.4611 |
| PC3 | 0.4041 | 0.4128 | 0.4075 | 0.4162 |
| PC4 | 0.3949* | 0.4330 | 0.4374 | 0.4391 |
| PC5 | 0.4766 | 0.4072* | 0.4363 | 0.4657 |
| PC6 | 0.4501 | 0.4870 | 0.4443 | 0.4670 |
